# Can the First Web Space Angle Be Predictive of Carpal Tunnel Syndrome?

**Published:** 2019-02

**Authors:** Cuma UZ, Ebru UMAY, Ibrahim GUNDOGDU, Aytul CAKCI

**Affiliations:** Physical Medicine and Rehabilitation Clinic, Ankara Diskapi Yildirim Beyazit Education and Research Hospital, University of Health Science, Ankara, Turkey

**Keywords:** Carpal tunnel syndrome, Electrophysiology, Anthropometry

## Abstract

**Background:**

Carpal tunnel syndrome (CTS) is the most frequent entrapment neuropathy in the upper limb. Although more objective methods for assessment have been reported in literature, there is a lack of evidence concerning the best methods for assessment of CTS. This study aimed to investigate whether there was a difference in the first web space in patients with different severities of CTS in relation to healthy controls as easy screen method.

**Methods:**

This prospective controlled trial was conducted on 126 patients at the Physical Medicine and Rehabilitation Clinic, Ankara Diskapi Yildirim Beyazit Education and Research Hospital, University of Health Science, Ankara, Turkey, from January 2016 to January 2018. Hand grip and pinch strength of patients were determined. Also, first web angle were measured by goniometer. Patients were divided into 3 CTS groups as electrophysiologically: “mild: group 1”, “moderate: group 2” and “severe: group 3”. Patient and healthy groups were compared in terms of the evaluation parameters. Comparisons were also made between these groups.

**Results:**

There was significant reduction in hand strengths and first web angle in patient groups compared to healthy groups (*P*<0.05). Moreover, the first web angle was significantly different between the CTS groups (*P*= 0.001). The cut-off value for CTS was <38.5°.

**Conclusion:**

The possibility of CTS can be evaluated by measuring the first web space angle with a simple goniometer as a easy and in-expensive method in outpatient clinics.

## Introduction

Carpal tunnel syndrome (CTS) is the whole of the symptoms that occur when the median nerve is trapped in the carpal tunnel. Carpal tunnel syndrome (CTS) is the most frequent entrapment neuropathy in the upper limb (1). If this nerve compression is prolonged in the carpal tunnel, the patient will develop sensory symptoms such as paresthesia (tingling or itching sensation), numbness, pain and motor symptoms such as stiffness, and also weakness of hand capabilities (2,3). The occurrence of the above symptoms would affect the performance and efficiency of the patient’s ability in performing daily tasks (4). The most daily hand-related tasks require the ability to produce, maintain and regulate pinch force to elaborately manipulate objects (5). It is common that the task not only requires the person to induce a minimum force to enable it to be carried out, but also to maintain that minimum force for a certain duration to fully complete the required task(5,6). For these reasons, CTS intervention is required before any worsening of symptoms, the development of muscle atrophy and diminished functionality.

Although there are many factors such as metabolic/endocrine, inflammatory and non-inflammatory structural diseases and trauma in CTS etiology, mostly the event is idiopathic(7,8). The idiopathic group of CTS is involved in the etiology of repetitive activities and stresses of current work (such as factory workers, secretaries and housewives) leisure time activities and hobbies. Because of many factors in the idiopathic group, the diversity of treatment is high whereas the response to treatment is low. Therefore, CTS is the most expensive upper-extremity musculoskeletal disorder when estimating the cost of medical care all over the world primarily due to surgical releases (9). For this reason, it is important to identify CTS diagnosis related factors.

Although more objective methods for assessment (such as electrophysiological, clinical evaluation, ultrasonographic and anthropometric methods) have been reported in literature, there is a lack of evidence concerning the best methods for assessment of CTS (3,6,10–13). Although these studies provide valuable information, in most clinical practice they are complex, expensive, time-consuming and require equipment. In this case we should ask ourselves the following question: In outpatient clinics, can we predict the possibility of CTS with simple methods?

In order to search for an answer to this question, we investigated whether there was a difference in the first web space in patients with different severities of CTS in relation to healthy controls.

## Materials and Methods

This prospective controlled trial was conducted on 126 patients at the Physical Medicine and Rehabilitation Clinic of our hospital between January 2016 and January 2018. Patients who were aged between 18 and 65 years, with a diagnosis of carpal tunnel syndrome (CTS) confirmed using electroneuromyography (EMG) upon referral to the EMG laboratory with pain, paresthesia and weakness symptoms in the hands. Sex, age, hand dominancy and body mass index-matched (BMI) healthy volunteers (n=104) with normal EMG and without CTS symptom and findings, that occurs relatives of patients and hospital staff, included as the control group.

Subjects, aged under 18 or older than 65 years of age, with non-dominant hand involvement, inflammatory, autoimmune, endocrine, metabolic, severe renal disease, central nervous system disease, cervical disc herniation, polyneuropathy, peripheral entrapment neuropathy, a history of hand trauma or surgery, hand and finger osteoarthritis, anatomic variation, ganglion cyst, tenosynovitis or tendinitis on the hands and subjects during pregnancy or lactation were excluded.

All subjects have provided written, informed consent for the study which has been approved by the local Ethical Committee.

### Elecrophysiological Evaluations

Electrophysiological evaluation that applied to all subjects, was conducted by a single physical medicine and rehabilitation (PMR) specialist using a Medelec Synergy (Oxford, UK) 10 channel electroneuromyography device. According to the protocol defined by Oh et al. (14), median peak sensory conduction velocity (wrist to second finger) slower than 41.25 m/sec, mixed nerve peak conduction velocity (palm to wrist) slower than 34 m/s, and / or prolongation of distal motor latency of median nerve (wrist to abductor pollicis brevis muscle) (>3.6 msec) were evaluated as CTS.

Slowed on median sensory and mixed nerve conduction velocity with normal sensory action potential amplitude was interpreted as mild CTS. In addition to these findings, if the median nerve distal motor latency was prolonged, it was interpreted as moderate CTS. In sensory and motor conduction studies, it was assessed as severe CTS if the sensory action potential was absent and/or compaund motor action potential was decreased.

### Demographics and Disease Characteristics

Demographic characteristics including age, gender, educational status, occupation, dominant hand, height, weight, and BMI of the all subjects were recorded. Educational status was classified as “illiterate”, “less than 5 years”, “5 years”, “8 years”, “11 years” and “over 11 years”.

### Clinical Evaluation

Measurements of maximal hand grip strength were determined by means of the Jamar-Dynamometer. To standardize the results, the hand grip strength test was conducted in the same position for each subject (in sitting position, shoulder in full adduction and elbow at 90° flexion, wrist in semipronation, thumb in an upward pointing position). The assessments were taken three times consecutively and the average values were taken in pound. Finger grip was assessed by pinchmeter in three separate positions (lateral, palmar, fingertip) (15).

### Anthropometric Assessments

**Fig. 1:**
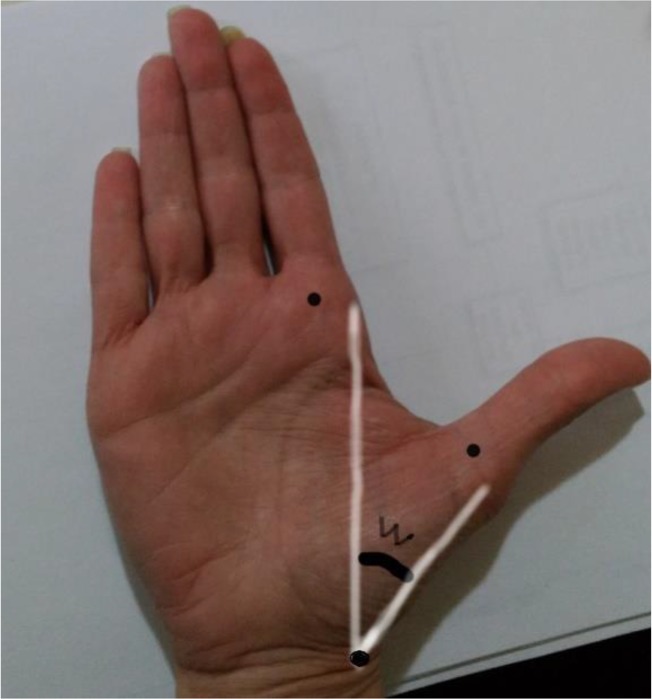
The first web space angle measurement method Center of metacarpophalangeal joint, W: the first web space angle

The hands of the subjects were placed so that the dorsum of the hand would come into contact with the table entirely and the volar aspect of the hand pointing upward. The thumb was positioned in maximum radial abduction and no passive thumb stretch was attempted as well as the following steps were followed: Three areas were marked-i) radial styloid process, ii) radial border of the MCP joint of the index finger iii) radial border of at the MCP joint of the thumb. The three marks were connected with two lines. The angle formed by the two lines was measured by means of a goniometer with the axis placed at the web space mark and the two arms at the index and thumb marks (Fig. 1) (16,17).

### Study Protocol

All evaluations were carried out within the same day. Electrophysiological evaluations were performed by the same PMR specialist blinded to group information. Strength measurements were performed after anthropometric assessments by an other PMR specialist blinded to ENMG evaluations.

### Comparisons

Patient group and healthy volunteers were compared in terms of the evaluation parameters. Patients were divided into 3 groups according to ENMG evaluation results: “mild CTS: group 1”, “moderate CTS: group 2” and “severe CTS: group 3”. Comparisons were also made between the these groups.

### Statistical Analysis

SPSS 25.0 (Chicago, IL, USA) was used in the analysis of the data. In descriptive statistics, the data were expressed as mean ± standard deviation for continuous variables, and as frequencies and percentages (%) for nominal variables. The Kolmorov-Smirnov test was used to determine whether or not they exhibited normal distribution for the continuous variables. The significance of the differences between the groups in terms of normal undistorted continuous variables were analyzed by the Student *t* test and the ANOVA test, and the significance of difference for nominal variables was analyzed using the Fisher exact test. Logistic regression analysis was applied for significant correlations. Receiver operating characteristic (ROC) curves were formed for the cut-off value of the angle between patients and healthy volunteers. Area under the curve (AUC), cut-off point, sensitivity and specificity values were calculated. The results were considered statistically significant when p <0.05.

## Results

The mean age of all 230 subjects in the present study was 51.37 ±11.05 years, 213 (92.6%) were female and 17 (7.3%) were male and 222 (96.5%) subjects had right hand dominancy as well as the most of the patients were housewives (n=194, 84.3%).

The distribution of demographics of the patients and healthy volunteers is shown in Table 1. Patient groups and healthy volunteers were similar in terms of demographic characteristics. (*P*>0.05). The grip and pinch strengths of the patient group and healthy volunteers and the distribution and comparison of the anthropometric evaluations are presented in Table 2. There was significant difference in all grip and pinch strengths and first space web angle between the patient groups and healthy volunteers (*P*<0.05).

**Table 1:** The distribution of demographic characteristics of the patients and healthy groups

***Variable***	***Patient group*** ***n=126*** ***mean±SD, n(%)***	***Healthy group*** ***n=104*** ***mean±SD, n(%)***	***P***
Age (yr)	53.28±14.87	50.62±9.18	0.119
Gender			
Female	115 (91.3)	98 (94.2)	0.376
Male	11 (8.7)	6 (5.8)	
Educational status			
Illiterate	7 (5.6)	4 (3.8)	
Under 5-years	24 (19.1)	15 (14.4)	
5-years	49 (38.8)	31 (29.8)	0.127
8-years	30 (23.8)	37 (35.6)	
11 years	11 (8.7)	10 (9.6)	
More than 11 years	5 (4.0)	7 (6.8)	
Occupation			
Housewife	107 (84.9)	87 (83.6)	
White collar	9 (7.1)	11 (10.6)	0.714
Blue collar	6 (4.8)	5 (4.8)	
Student	4 (3.2)	1 (1.0)	
Dominant hand			
Right	123 (97.6)	99 (95.2)	0.326
Left	3 (2.4)	5 (4.8)	
BMI (%)	31.66±3.08	31.29±3.28	0.761

SD: Standard deviation, BMI: body mass index, White collar

Blue collar

**Table 2:** The grip and pinch strengths of the patient group and healthy volunteers and the distribution and comparison of the anthropometric evaluations

***Variable***	***Patient group*** ***(n=126)*** ***mean±SD***	***Healthy group*** ***(n=104)*** ***mean±SD***	***P***
Grip strenght (pound)	18.87±7.79	22.06±9.39	0.015
Lateral pinch (pound)	7.50±2.20	9.26±4.76	0.022
Palmar pinch (pound)	6.83±1.99	8.53±3.68	0.017
Fingertip pinch (pound)	5.68±3.48	6.46±3.15	0.041
First web space angle (°)	35.38±7.78	62.65±24.82	0.001

SD: Standard deviation

Patients were divided into 3 groups according to EMG results. According to this; patients were classified as mild CTS group 1 (n = 27, 21.4%), moderate CTS group 2 (n = 41, 32.5%) and severe CTS group (n = 58, 46.1%). The distribution of grip and pinch strengths and web angle values according to the groups is present in Table 3.

**Table 3:** The distribution of grip and pinch strengths and web angle values according to the groups

***Variable***	***Group 1*** ***n=27*** ***mean±SD***	***Group 2*** ***n=41*** ***mean±SD***	***Group 3*** ***n=58*** ***mean±SD***	***P***
Grip strenght (pound)	19.15±8.62	18.56±6.54	17.80±6.56	0.916
Lateral pinch (pound)	8.75±1.65	7.51±2.28	7.32±2.13	0.485
Palmar pinch (pound)	7.37±1.18	6.87±2.39	6.76±1.80	0.834
Fingertip pinch (pound)	6.12±1.37	5.87± 1.36	5.54±1.56	0.537
First web space angle (°)	40.27±7.41	37.29±10.81	33.46±7.27	0.001

SD: Standard deviation

**Fig. 2:**
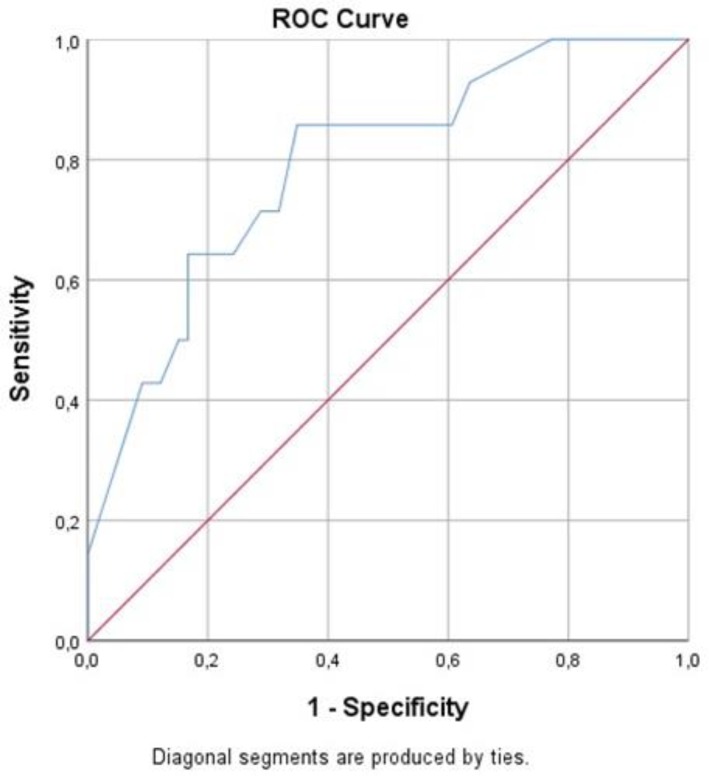
Receiver Operating Characteristic (ROC) curve analysis results

Only the web angle was significantly different between the groups (*P*= 0.001). In subgroup analysis; the difference between all groups was significant (*P*= 0.023 between groups 1 and 2, *P*= 0.001 between groups 1 and 3, *P*= 0.020 between groups 2 and 3). Regression analysis showed that the web angle was a factor associated with the severity of CTS (95% CI: 0.075–0.443, *P*= 0.001). According to the ROC curve analysis of the web angle of CTS patients and healthy control, the cut-off value for CTS was <38.5° and the sensitivity and specificity were evaluated as 82.9% and 77.3%, respectively. (AUC 0.792, 95% CI: 0.665–0.919) (Fig. 2).

## Discussion

Although numerous diseases (rheumatoid arthritis, ganglion cysts, bony protrusions, muscle and tendon anomalies, which reduce the volume in the carpal tunnel; diseases that facilitate compression such as diabetes mellitus, hypothyroidism and personal reasons such as overuse of the hand) have been implicated as the cause of carpal tunnel syndrome (CTS), most of them are idiopathic(2,7,18).

The clinical symptoms and physical examination findings in patients with CTS are recognised widely and various treatments exist, including non-surgical and surgical options. Early diagnosis of CTS increases the responsiveness of treatment. Despite these advantages, there is a paucity of evidence about the best approaches for assessment of carpal tunnel syndrome(10). More objective methods for assessment, including electrodiagnostic testing and nerve imaging, provide additional information about the extent of axonal involvement and structural change, but their exact benefit to patients is unknown. In addition, in clinical practice, electrophysiological evaluation of almost all CTS patients is time consuming and expensive. Therefore in literature; there are lots of publications which have been based on evaluation of clinical signs, symptoms and possible risk factors to diagnose CTS (19–21).

Anthropometric evaluations are also methods that have been studied over the past years and the relationship between hand structural features and CTS (6,11,22–26). According to these studies, we can predict pathophysiological changes in the carpal tunnel in terms of slower capillary circulation, hypoxia of nerve fibres, oedema and increased pressure inside the carpal tunnel, and consequently reduced median nerve conductivity. In anthropometric studies conducted in literature; it has been investigated as to whether or not features such as hand length, wrist width and wrist ratio were related to CTS in relation to healthy controls, but the results were found to be contradictory(22–26). As a result, it has been reported that other personal factors such as hand use status and age, which facilitate compression, may also affect these factors.

In recent years, studies have shown that there is a sensorimotor dysfunction of the median nerve, particularly concerning the thumb and index finger in CTS (6,11). In these studies, it was reported that these two fingers had changes in the directions of movement and contact areas during movement in patients with CTS. It has also been reported that the pinch strength of these two fingers due to the involvement of motor branches also decreases.

However, these studies reveal only fixed anthropometric features and do not show the characteristics of each person’s hand, such as skin and muscle changes that may arise with the use of one’s hand. In addition, they were unable to reveal the variable pressure differences in the carpal tunnel due to overuse. Changes in hand or even hand skin may occur with any increase in hand use (27). The hand is an organ that is involved in practically all our daily activities, thus presenting a variety of functions, and to function perfectly it needs complete harmony between and among the various constituent tissues.

Based on these studies, we investigated whether there was a difference in the first web space which is a method that can be applied simply and outpatient setting, in patients with different severity of CTS. As a result of this study, it was shown that this space decreased significantly compared to healthy individuals. Moreover, as the severity of the CTS increased, it continued to decrease significantly. In addition, it was found that sensitivity of 38.5° web space is 82.9% and specificity is 77.3% for CTS diagnosis.

The first web space between the thumb and index finger is formed by abduction of the 1st finger. Abduction of the thumb is formed by the contraction of the abductor pollicis brevis (APB) muscle, which is innervated by the median nerve, and adduction of the thumb; the contraction of the adductor pollicis (AP) muscle, which is innervated by the ulnar nerve (28,29). The nerve conduction study of the motor branch of the median nerve is provided by the APB muscle. Median nerve compression affects firstly sensory branches, than motor fibers are also affected by increased compression3. In this regard, both the reduction of abduction force, as well as muscle imbalance between abduction and adduction muscles due to different neural innervation of that are likely to lead to a reduction of the web space. For these reasons; we think that the increase in severity in CTS may explain the decrease in the web space. This is supported by the fact that grip and pinch strengths was lower in the CTS group than in the normal group. But interestingly, even in patients with mild CTS, which affected only the sensory nerves there was significantly less web space than in healthy controls.

In studies, during the second finger’s repetitive and prolonged movements, even if there is no forced activity, the lumbrical muscles of the index finger have been shown to be able to occupy a part of the carpal tunnel in CTS patients and reported that lumbrical muscles could compress the median nerve (11, 28). The first lumbrical muscle which provides pinch movement between thumb and index fingers, has been shown to be responsible for an increase in pressure within the carpal tunnel as finger flexion increases (29).

Most subjects of our study were middle-aged housewives that used just their hands all day and we believe that due to this there may have been an intermittent but constant increase in pressure. In previous studies, the severity and the rate of neuropathic changes correlate not only with the degree and duration of the compression, but also with the acuity of pressure elevation(3,6). Moreover, some studies have shown that with compression of the median nerve caused by intermittent pressure increases, it may diminish force-coordination of grip and pinch, neuropathic effects on the ability to skillfully move the digits can also limit dexterous manipulation of objects. That is, the muscle function can be affected in the early period (11). When we synthesize our own results with those results, we think that muscle imbalances are present even in the early stages of CTS, and as a reflection of this, the first web space is narrowing. Moreover, we think that muscle imbalance and coordination are progressing parallel to the severity of CTS.

There were some limitations of the current study. One of them was that the majority of our patient group were housewives. For this reason, our results can be considered as not covering the whole population. Therefore, we believe that large-scale studies involving occupations such as computer active or factory workers will provide a better understanding of our results.

## Conclusion

In CTS patients, neuropathic changes occur from an early stage, and as a reflection of this, the first web space narrows. Before electrophysiological tests are performed, the possibility of CTS can be evaluated by measuring the first web space angle with a simple goniometer as a non-invasive and inexpensive method in outpatient clinics.

## Ethical considerations

Ethical issues (Including plagiarism, informed consent, misconduct, data fabrication and/or falsification, double publication and/or submission, redundancy, etc.) have been completely observed by the authors.
